# Purification of ethanol for highly sensitive self-assembly experiments

**DOI:** 10.3762/bjnano.5.139

**Published:** 2014-08-12

**Authors:** Kathrin Barbe, Martin Kind, Christian Pfeiffer, Andreas Terfort

**Affiliations:** 1 Institut für Anorganische und Analytische Chemie, Goethe-Universität Frankfurt, Max-von-Laue-Straße 7, 60438 Frankfurt am Main, Germany; 2 Fachbereich Chemie, Philipps Universität Marburg, Hans-Meerwein-Straße, 35032 Marburg, Germany

**Keywords:** ethanol, gold nanoparticles, purification, self-assembled monolayers, solvent

## Abstract

Ethanol is the preferred solvent for the formation of self-assembled monolayers (SAMs) of thiolates on gold. By applying a thin film sensor system, we could demonstrate that even the best commercial qualities of ethanol contain surface-active contaminants, which can compete with the desired thiolates for surface sites. Here we present that gold nanoparticles deposited onto zeolite X can be used to remove these contaminants by chemisorption. This nanoparticle-impregnated zeolite does not only show high capacities for surface-active contaminants, such as thiols, but can be fully regenerated via a simple pyrolysis protocol.

## Introduction

Thirty years after their introduction [1–3], self-assembled monolayers (SAMs) of organothiolates have matured to an established tool for surface modification [4], e.g., as etch resist for microfabrication [5–6], as support for molecular systems like metal-organic frameworks or biomolecules [7], or for the tuning of electronic properties of metal surfaces [8–12], to mention just a few. One reason for the popularity of SAMs is their ease of preparation: Simple immersion of the substrate, typically gold, silver, or palladium, into micro- or millimolar solutions of the respective organic thiols [2–3] or disulfides [1] yields the monolayers as well defined systems. At least for the gold/thiolate system, it has been claimed that the thiolate units bind strongly enough to replace most contaminants, making a thorough cleaning of the substrate unnecessary [4]. While this seems to be true for long-chained *n*-alkanethiols, which have been used (in combination with hydrogen plasma) to clean gold substrates [13], it has been reported for other systems that contaminants remain within the layer system [3,14–15]. In some cases, even the constituents of a fully formed monolayer could be removed by competing molecules [16–21]. It therefore seems to be advisable not only to use very clean gold substrates but also to avoid any contaminants. A solvent typically considered to be clean is ethanol, which therefore is very popular for the formation of thiolate monolayers. Other reasons for its popularity are its low toxicity and reasonable price. It dissolves most thiols to a suitable concentration (micro- to millimolar), and does not remain within the monolayer [4].

On the other hand, ethanol is typically produced by fermentation of biomass [22]. In this process, driven by yeasts like *Saccharomyces cerevisiae*, a plethora of sulfur-containing compounds is produced, among them hydrogen sulfide, dimethyl sulfoxide, short-chained organothiols like methanethiol or ethanethiol, thioacetates (methyl thioacetate, ethyl thioacetate), sulfides (e.g., dimethyl sulfide) and disulfides (e.g., dimethyl disulfide) [23], all of which exhibit a certain affinity to gold: Hydrogen sulfide is known to form S–Au bonds on gold surfaces at room temperature, thereby releasing hydrogen [24]. Organic thiols [4], thioacetates [25] and disulfides [26] form thiolates on gold while organic sulfides [27] and dimethyl sulfoxide [28] usually remain intact when binding to gold surfaces. Industrial cleaning procedures like distillation are applied to remove these contaminants, yet even very low concentrations of remaining sulfur-containing molecules are sufficient to cover a gold surface: To form a dense monolayer, only about 1 nmol cm^−2^ of surface-active molecules is needed [4]. Even highly pure commercially available ethanol, such as the one produced for high-performance liquid chromatography (HPLC), suffers from the drawback that the purification procedure is not specific for sulfur-containing contaminations. While these contaminations might only play a minor role in most cases of SAM formation, where thermodynamic equilibrium can be approached by long exposure times, it has been found that for kinetic studies even very minor contaminations can significantly affect the measurements [29]. Of the different time-resolved methods to determine monolayer formation, surface-plasmon resonance (SPR) [30–31] and a resistivity sensor developed by Bohn et al. [29,32] show the highest sensitivity and accuracy. The latter sensor is based on the change of the electrical resistance of a very thin metal film upon adsorption as described by the Fuchs–Sondheimer theory [33–34]. Since this is a purely interfacial phenomenon, this kind of sensor is equally sensitive for small and larger molecules, in contrast to SPR, second-harmonic generation (SHG) spectroscopy [35] and quartz-crystal microbalance (QCM) experiments [36].

To employ this extremely sensitive technique for the determination of adsorption kinetics, we tested ethanol of several commercial qualities and had to find that even the most pure ethanol samples did not lead to satisfactory results (see below). We therefore set out to develop a method for the complete removal of impurities with an affinity for gold surfaces. Since we could not pin-point a single class of molecules to be specifically removed from the solvent, we rather wished to use the affinity of these impurities to gold to remove them. For this, the use of high-area gold surfaces, such as fine gold powder, would be optimal but also prohibitively expensive. As an alternative, Bohn et al. used copper powder, followed by distillation, to obtain ethanol sufficiently pure for their kinetic studies [29]. In our hands, this method had only limited success, presumably since copper powder is often treated with surface active compounds to suppress oxidation. In addition, not all the compounds with an affinity to gold could be removed by using copper, presumably due to slight differences in the reactivity of gold and copper towards these compounds. We therefore decided to use gold nanoparticles (NPs), which have a very high surface/weight ratio, to obtain purified ethanol. For this, the NPs were immobilized onto an inert, porous support, zeolite, to facilitate the handling and to suppress coagulation. These hybrid systems were characterized using (scanning) transmission electron microscopy (TEM/STEM) and energy-dispersive X-ray (EDX) spectroscopy. The cleaning capacity of the immobilized NPs and their recyclability depending on the preparation conditions were tested by exposing them to solutions of a test substance (dodecanethiol). As proof of concept, the applicability for ethanol purification was demonstrated by employing the sensoric principle developed by Bohn et al. [29,32].

## Results and Discussion

**Figure 1:**
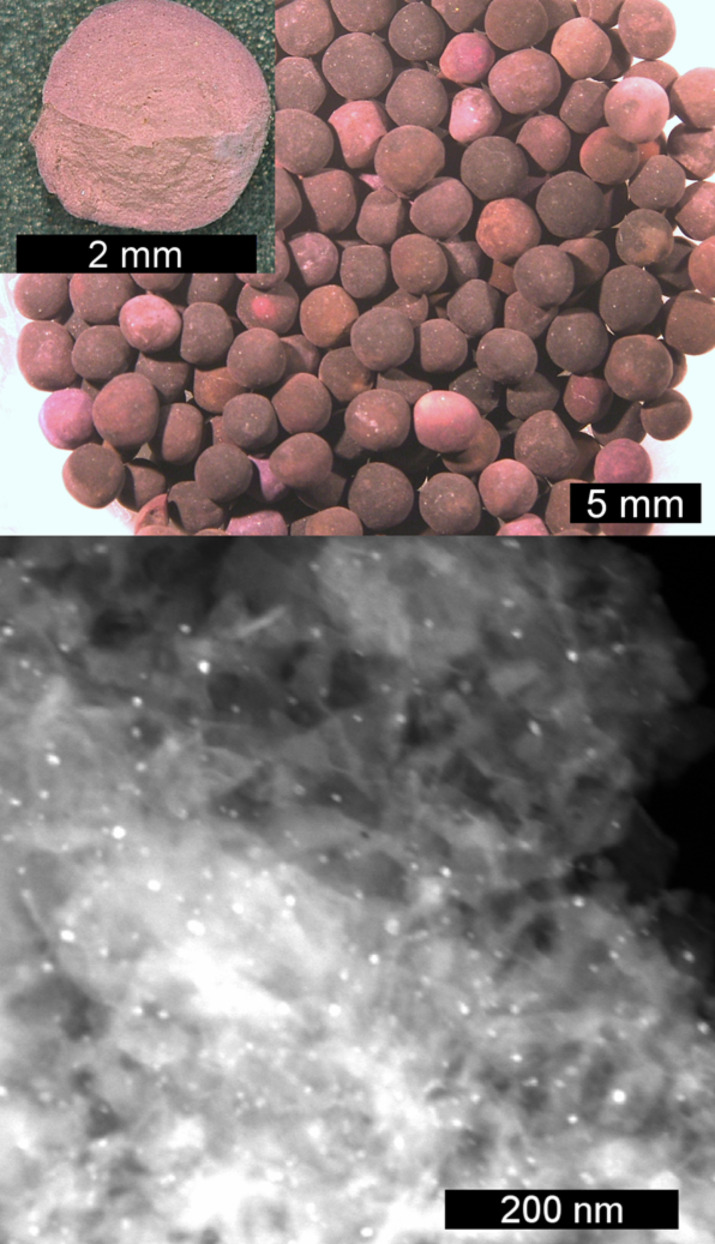
Top: Picture of molecular sieve beads (zeolite X) loaded with gold-NPs, from a batch used for ethanol purification, taken with an optical microscope. The inset shows a micrograph of a bead with a fresh breakage at the lower side, the color of which indicates that the NPs are located not only on the surface but also within the beads. Bottom: Scanning transmission electron micrograph of gold-NPs (bright spots) immobilized on zeolite X.

Several methods exist to deposit noble metal nanoparticles onto porous substrates, the most popular being the adsorption and decomposition of a precursor material, such as a salt of the respective noble metal. This technique is widely used to produce catalysts in industry [37]. We decided to use the most common gold compound, tetrachloridoauric acid (HAuCl_4_), as starting material. This material can be readily adsorbed onto commercial zeolite X by soaking its solution in ethanol/water into the pores of the aluminosilicate. To facilitate the handling, we used the beaded material, commercially available as ‘molecular sieve’, instead of the pure, powdered zeolite. The color of these beads changed from off-white to yellow after immersion into the HAuCl_4_ solution and to violet upon pyrolysis in the tube furnace, indicative for the formation of the Au-NPs with their characteristic Mie scattering behavior [38]. Typical samples of zeolite grains after formation of the gold-NPs are displayed in Figure 1, top.

While the somewhat heterogeneous coloration of the outside of the beads suggests an uneven distribution of the gold, a break-up of a typical bead shows a uniform coloration, suggesting an even distribution of the gold-NPs. EDX spectra (compare Figure S1 in Supporting Information File 1) of the Au-NP loaded beads show signals of Al, Si, O (due to the zeolite material) and Au, supporting the successful loading. STEM images were recorded to not only learn about the distribution of the NPs, but also about their density and their size. As can be seen in Figure 1, bottom, even on this scale the particles seem to be evenly distributed, although their density is not very high. It can also be seen that most of the NPs have a diameter of about 5 nm, but some are larger (up to 20 nm). Since this size significantly exceeds the diameters of the pores of the zeolite, it might be concluded that the NPs destroyed the pores during growth. Anyway, we rather believe that the NPs were formed at grain boundaries, which exist in abundance and where size restrictions apply less rigidly.

**Figure 2:**
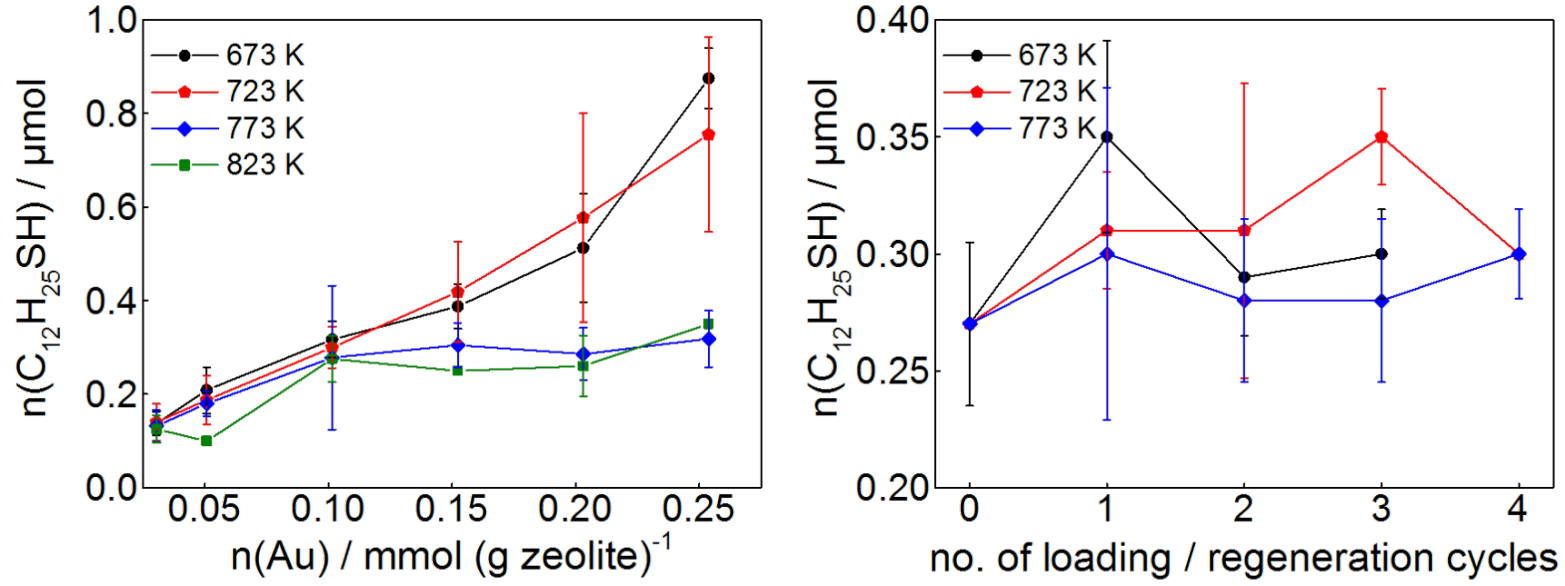
Left: Absolute uptake of dodecanethiol in dependence of the molar amount of gold per 1 g of NP-impregnated zeolite. As can be seen, the pyrolysis temperature plays an important role for the uptake capacity. Right: Thiol uptake capacity of 1 g of NP-impregnated zeolite (0.1 mmol Au/g), recycled at different temperatures.

The ability of the zeolite-supported gold-NPs to remove thiols and other surface-active impurities from ethanol was tested by varying the gold load at the zeolite as well as the gold-NP preparation temperature. Dodecanethiol was used as test substance for these chemisorption experiments because it binds effectively and fast to gold surfaces [3]. In a typical test, one gram of the zeolite loaded with different amounts of gold-NPs was stirred into solutions of dodecanethiol in ethanol. After ten minutes, the solution was tested for remaining thiol using Ellman’s reagent. By increasing the concentrations of the solutions in small steps until the test became positive (formation of yellow color) the maximum amount of adsorbable dodecanethiol was determined. In Figure 2, left, the dodecanethiol uptake capacity per gram of NP-impregnated zeolite material (given in mmol dodecanethiol) is presented for materials with different gold content and prepared at different temperatures.

As evident from Figure 2, the uptake capacity of samples pyrolyzed at all temperatures behaves similarly for the samples with low gold loadings (≤0.1 mmol Au/g). At higher loadings, the uptake capacities of samples heated to higher temperatures (773 K and 823 K) does not increase any more with increasing gold content. This is probably due to partial melting and subsequent coalescence of the gold-NPs [39], which results in a smaller surface to bulk ratio, i.e., a lower fraction of thiol binding sites. In contrast to this, the dodecanethiol uptake capacity of zeolite-supported gold-NPs pyrolyzed at lower temperatures (673 K and 723 K) increases throughout the whole range of gold loading. Yet for all temperatures, a doubling of the gold loading leads to less than a double dodecanethiol uptake capacity. We speculate that higher gold loadings lead to formation of gold-NPs with a smaller surface to bulk ratio. Thus, an effective and cheap purification can best be achieved with low gold per zeolite mass ratios.

It is well known that sulfur-species adsorbed on gold can be removed by heating to moderate temperatures [40–41], opening the opportunity to recycle the NP-impregnated zeolite for repeated use. To test the recyclability, samples with 0.1 mmol nanoparticulate gold per gram were prepared at 773 K, fully loaded with dodecanethiol and heated to different temperatures in a stream of nitrogen. In Figure 2, right, the dodecanethiol uptake capacities of zeolite-supported gold-NP samples after regeneration at different temperatures are plotted against the number of loading-regeneration cycles. The thiol uptake capacities of the three samples remain essentially constant, though the data show some scattering, which at least partially is due to the fact that the uptake was measured in a stepwise manner. The samples prepared and recycled at 723 K maintain somewhat greater uptake capacities than the other two series of samples. Based on this finding, we established the use of zeolite impregnated with 0.1 mmol of nanoparticulate Au per gram of material at 773 K for further use in our laboratory. This material advantageously becomes regenerated at 723 K without any loss of thiol adsorption capacity.

**Figure 3:**
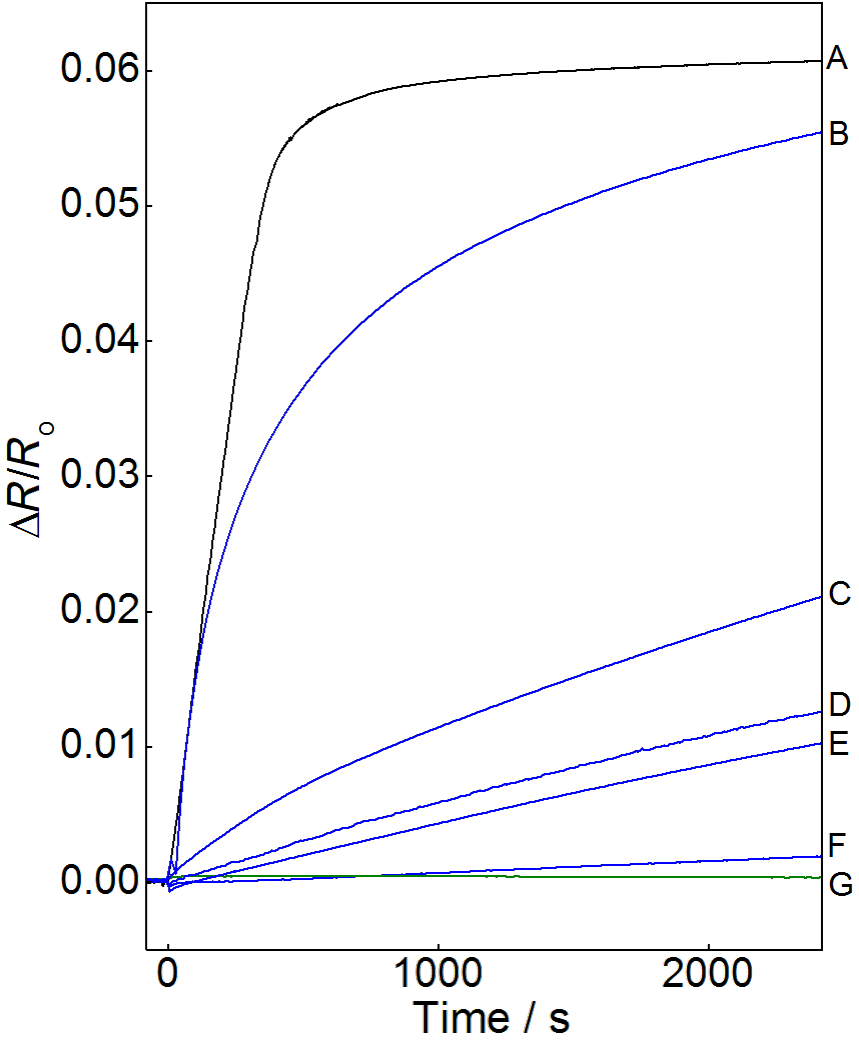
Relative change of resistivity of a thin gold film sensor upon immersion into ethanol species of different purity grades at 298 K. The gold surface was immersed into the liquids at 0 seconds. The successive resistivity change indicates chemisorption of contaminant species (or in case of curve A, deliberately dissolved organothiols) in the ethanol samples onto the gold surface (A: 1 µM hexadecanethiol solution in ethanol of type G (see below); B: ethanol, technical grade, 99%, denatured with 1% petrol ether; C: ethanol of type B, distilled before measurement; D: ethanol “pro analysi”; E: ethanol “for spectroscopy”; F: ethanol “HPLC grade”; G: ethanol of type C, purified twice with zeolite-supported Au-NPs).

With this material at hand, we set out to purify ethanol to become suitable for kinetic measurements. As an assay, we used the sensoric principle developed by Bohn et al. [29,32], based on the resistivity change of thin gold films upon chemisorption of molecular films. For a selected class of compounds, such as alkanethiols, a very good linear correlation between the increase of resistivity and the surface coverage Θ has been established [29,32]. As an initial test, several commercial qualities of ethanol were checked for surface active compounds. As can be seen in Figure 3, curves B to F, even the best commercially available ethanol samples contain enough contaminants to deposit at least partial layers onto clean gold surfaces. It can be expected that these surface-active compounds compete with the thiols to be deposited resulting in SAMs of minor quality. Note that ethanol of technical quality (curve B, 99% certified purity) delivers surface-active compounds almost as quickly as a 1 µM solution of hexadecanethiol (curve A). Distillation of this sample results in a markedly smaller signal (curve C), indicating that a noticeable fraction of the surface-active contaminants has been removed, although the remainder still exceeds any acceptable limit for kinetic experiments.

To remove the surface-active compounds completely, the gold-NP impregnated zeolite material should be employed. Initial experiments focused on fixed-bed procedures in the hope to develop a simple filtration protocol. Unfortunately, even at low flow rates, the exposure time was too short to remove all the surface-active contaminants. Therefore, a prolonged contact between the gold-NPs and the ethanol was achieved by storing the ethanol over 3 weight % of the NP-impregnated zeolite in a clean flask under nitrogen and exclusion of light. The latter was necessary, since the action of light and air resulted in the formation of considerable amounts of ethyl acetate. When ethanol treated this way was tested for surface-active contaminations, the sensors became quickly destroyed (see Figure S2 in Supporting Information File 1). It turned out that the solvent ingested copious amounts of chloride ions from the zeolite, which in conjunction with air effectively dissolved the sensor metals. To remove these chlorine atoms, which obviously stem from the pyrolytic deposition of the Au-NPs (2 HAuCl_4_ → 2 Au + 3 Cl_2_ + 2 HCl), the ethanol had to be distilled before use, also helpful for removing tiny zeolite particles floating in the ethanol. It turned out to be advantageous to apply a sub-boiling setup instead of a regular still to eliminate the material transport by aerosol formation [42–43].

As can be seen in Figure 3, trace G, ethanol which has been purified by treatment with gold-NP impregnated zeolite and subsequent distillation is the only sample that causes no detectable resistivity change and thus can be assumed to be free from contaminant species that bind to gold surfaces. Note that for technical ethanol, the purification procedure had to be carried out twice: After the first run, the ethanol caused a resistivity change comparable to that of ethanol of spectroscopic quality (compare Figure S3 in Supporting Information File 1), while after the second cleaning step even this material did not contain any surface-active contaminations any more.

## Conclusion

Although ethanol is the standard solvent in formation of thiol-based monolayers, the natural content of surface-active contaminants has rarely been acknowledged. As we could demonstrate by applying the resistivity sensors established by Bohn et al. [29,32], even the best commercially available ethanol qualities contain enough impurities to induce noteworthy conductivity changes, which in turn indicate the occurence of chemisorption processes. While it cannot be excluded (but also not be concluded) that these chemisorbed impurities can become replaced by the desired thiols, their presence can significantly hamper, e.g., kinetic experiments or the formation of high-purity monolayers. In case of only weakly adsorbing thiols, such as adamantane thiols [18], strong interference can be expected.

The idea to use the very same metal in a highly disperse form to remove these contaminants led to the application of gold-NPs on a highly porous support, zeolite. We could demonstrate that this material can conveniently be prepared by an impregnation/pyrolysis route. Optimization of the gold content and the heating temperature led to a stable material that can easily be handled – and even more important – recycled without loss of activity. In fact, this material has been reused in our lab for ethanol purification for dozens of cycles without any loss of performance. By storing the ethanol over this material, followed by distillation just before use, basically ethanol of any quality can be rendered free of surface-active impurities, although in the most severe cases two treatments were necessary. We believe that this simple and efficient protocol opens the door for the very sensitive measurements of monolayer formation kinetics as well as the production of high-quality SAMs.

## Experimental

### Chemicals

Tetrachloridoauric acid was either purchased from Merck KGaA, Germany, or prepared by dissolving gold powder in hot hydrochloric acid while a stream of chlorine was passed through. The zeolite beads (zeolite X, average pore diameter 10 Å) were obtained from Roth, Germany. Dodecanethiol, hexadecanethiol, and Ellman’s reagent (5,5'-dithiobis-(2-nitrobenzoic acid)) were purchased from Sigma-Aldrich. Technical ethanol (99%, denatured with 1% petroleum ether) was supplied by Brenntag, Germany. Ethanol “for spectroscopy” (Uvasol), “pro analysi”, and “HPLC grade” (LiChrosolv) were purchased from Merck KGaA, Germany.

### Preparation and regeneration of zeolite-supported gold NPs

To obtain zeolite-supported gold-NPs, 10 g of zeolite beads were soaked with 10 mL of HAuCl_4_ solutions (0.03–0.25 mol/L, depending on the desired Au amount) in aqueous ethanol (50%). The samples were then dried at 343 K for 1–2 h. Subsequently, NP formation was achieved by heating the beads in a tube furnace in a N_2_ stream at 773 K for 3 h. For regeneration, the zeolite-supported gold-NP samples were first dried at 343–363 K and then heated to 723 K for 2.5 h in a tube furnace in N_2_ stream.

### Characterization of zeolite-supported gold NPs by STEM and EDX

For transmission electron microscopy (TEM) investigations, samples of the zeolite-supported gold NPs were dispersed in ethanol using an ultrasonic bath. Afterwards they were sprayed onto carbon-coated copper grids using a modified UIS250v Hielscher sonifier [44]. Measurements were carried out with a FEI TECNAI F30 S-TWIN microscope working at 300 kV. Scanning transmission electron microscopy (STEM) images were acquired with a scanning unit by a FISCHIONE high angular annular dark field (HAADF) detector. Energy-dispersive X-ray (EDX) spectra were acquired with an EDAX detector and quantified by Emispec ESVision software.

### Determination of the thiol-uptake capacity of zeolite-supported gold-NPs

The ability of the zeolite-supported gold-NPs to adsorb thiols was investigated by exposing 1 g of each sample to different amounts (0.1–1.0 µmol, in increments of 0.05 µmol) of dodecanethiol in 5 mL of ethanol. After a period of 10 minutes, a part of the supernatant solution was transferred to a thin-layer chromatography (TLC) plate. After drying, the TLC plates were immersed into ca. 0.1 mM solutions of Ellman's reagent in acetone and exposed to gaseous ammonia. Residual thiol molecules in the ethanolic solutions were indicated by formation of yellow spots. The highest dodecanethiol amount that did not lead to a color change of the TLC plates was assumed to be the thiol-uptake capacity of the respective zeolite-supported gold-NP samples.

### Purification of ethanol using the zeolite-supported gold NPs

After distillation, 1 L of technical grade ethanol was stored under exclusion of light for 3 weeks in N_2_ atmosphere over 30 g of zeolite impregnated with 0.1 mmol Au per gram of zeolite (pyrolysis temperature of 773 K). To minimize the contamination of the ethanol by aerosol formation during distillation, the final distillation was carried out in a home-built sub-boiling apparatus [42] at about 348 K. In case of technical ethanol as starting material, this procedure had to be carried out a second time using freshly regenerated zeolite-supported Au-NPs to obtain fully purified solvent.

### Thin film sensors for chemisorption measurements

Following the protocol of Bohn et al. [29,32], thin gold films were produced by physical vapor deposition of gold onto glass supports. Since the sensitivity of the sensors inversely scales with the film thickness, typically thicknesses just above the level of the formation of dense films were chosen (ca. 25 nm). Adhesion of these films was promoted by prior evaporation of a chromium layer of 2 nm thickness. The resistivity of these films was determined by two-point measurements using an Integra Series 2700 digital multimeter (Keithley), which was connected to a computer using the ExeLinX-1A software (Keithley). The chemisorption measurements were performed by immersing the gold films into the respective samples maintained at 293.1 ± 0.1 K by means of a FP 40 thermostat (Julabo). To avoid contamination by the glassware, it was cleaned by 10% KOH/H_2_O containing H_2_O_2_ (ca. 10 mM), followed by rinsing with hot water, deionized water and ultrapure water (Millipore, 18.2 MΩ cm) before each experiment.

## Supporting Information

Supporting information includes an energy-dispersive X-ray (EDX) spectrum of gold-NP in a grain of zeolite-supported gold-NP material, thin gold film resistivity measurements of chloride-containing ethanol, and of technical grade ethanol not treated with gold-NP and once and twice purified using gold-NP.

File 1 Additional diagramms.
