# Supplementary material for: Purification of ethanol for highly sensitive self-assembly experiments
Source: Beilstein J Nanotechnol. 2014 Aug 12;5:1254–60. doi: 10.3762/bjnano.5.139 (PMC4142897; doi:10.3762/bjnano.5.139)
Supplement: File 1 — Additional diagramms. [file Beilstein_J_Nanotechnol-05-1254-s001.pdf]

Supporting Information

for

Purification of ethanol for highly sensitive self-assembly experiments

Kathrin Barbe[1], Martin Kind[1], Christian Pfeiffer[2] and Andreas Terfort*[1]

Address: [1]Institut für Anorganische und Analytische Chemie, Goethe-Universität Frankfurt, Max-von-Laue-Straße 7, 60438 Frankfurt am Main, Germany and [2]Fachbereich Chemie, Philipps Universität Marburg, Hans-Meerwein-Straße, 35032 Marburg, Germany

Email: Andreas Terfort - aterfort@chemie.uni-frankfurt.de

* Corresponding author

Additional diagramms

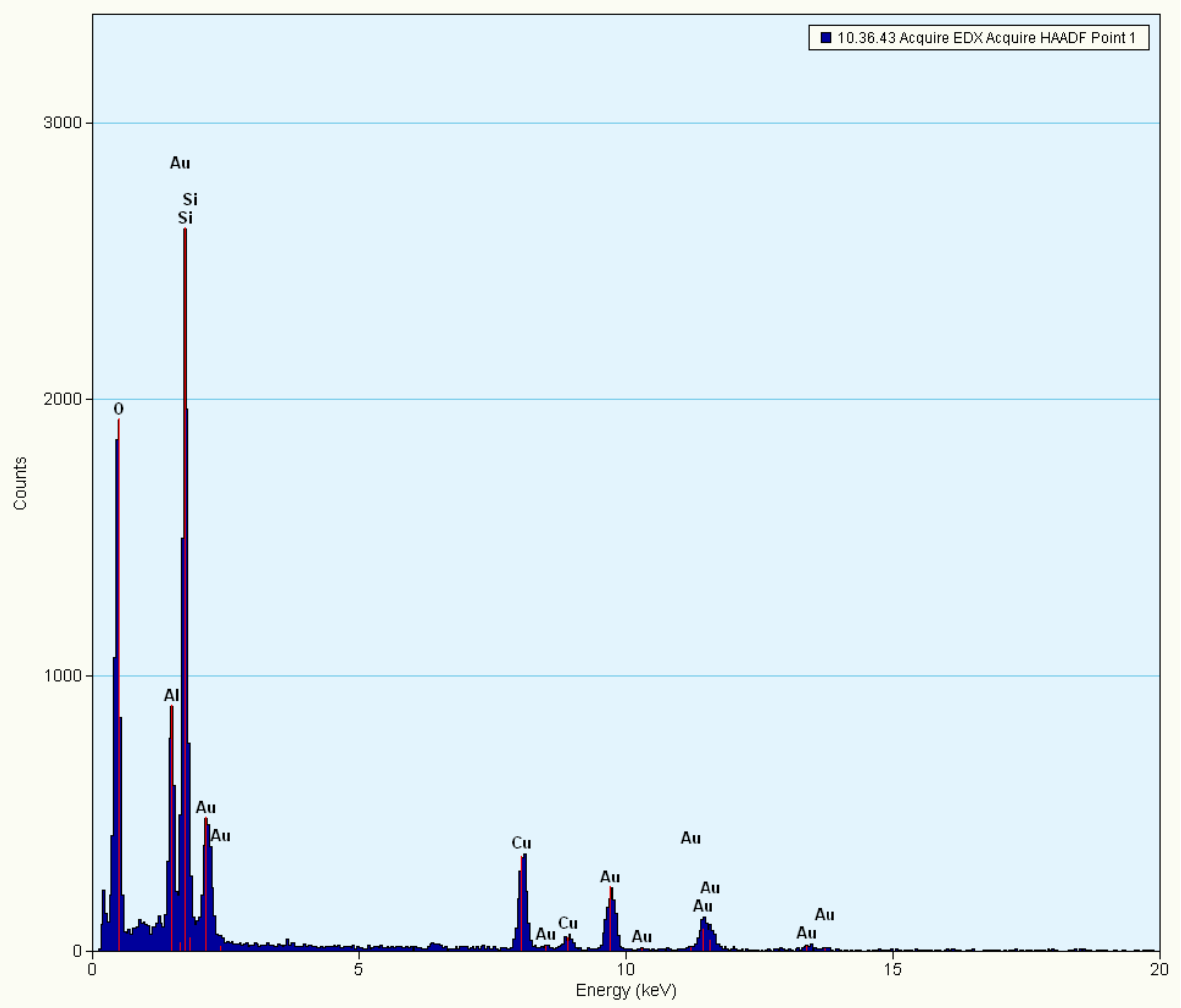

Figure S1: Energy-dispersive X-ray (EDX) spectrum of a gold-NP in a grain of zeolite-supported gold-NP material. Note that the Al, Si, O and Au signals are due to the sample while the Cu signal stems from the TEM grid.

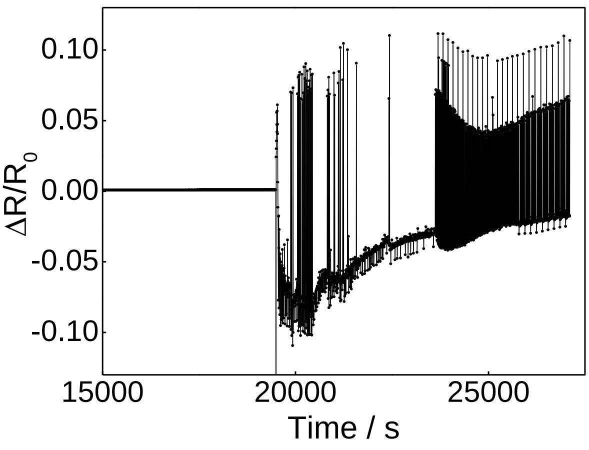

Figure S2: Relative change of resistivity of a thin gold film sensor upon immersion into ethanol technical grade, 99%, denatured with 1% petrol ether. In this case the ethanol was distilled prior to treatment with the gold-NP impregnated zeolites but not distilled afterwards. The scattering in the measured resistivity indicates leakage currents that are due to the presence of chloride ions.

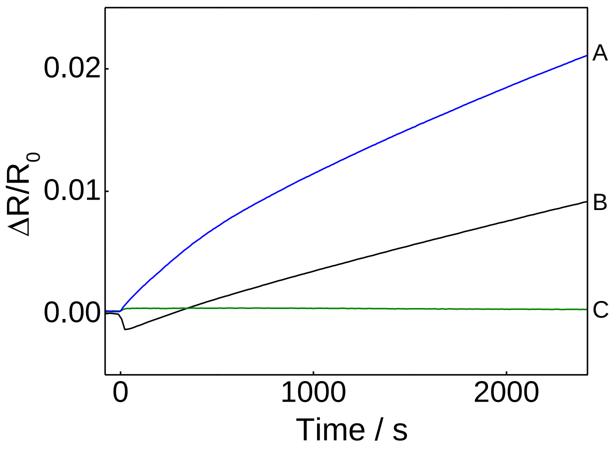

Figure S3: Relative change of resistivity of a gold film sensor upon immersion into ethanol species of different purity grades at 298 K. The sensor was immersed into the liquids at 0 seconds. The successive resistivity change indicates chemisorption of contaminant species in the ethanol samples onto the gold surface. For sample C, no chemisorption could be detected using this sensor system.

A: ethanol technical grade, 99%, denatured with 1% petrol ether, after distillation (curve identical to curve C in Figure 3)

B: ethanol of type A, cleaned with NP-impregnated zeolite and distilled

C: ethanol of type A, twice cleaned with NP-impregnated zeolite and distilled (curve identical to curve G in Figure 3)
